# Effects of High Hydrostatic Pressure on Bacterial Growth on Human Ossicles Explanted from Cholesteatoma Patients

**DOI:** 10.1371/journal.pone.0030150

**Published:** 2012-01-23

**Authors:** Steffen Dommerich, Hagen Frickmann, Jürgen Ostwald, Tobias Lindner, Andreas Erich Zautner, Kathleen Arndt, Hans Wilhelm Pau, Andreas Podbielski

**Affiliations:** 1 Department of Otorhinolaryngology, Head and Neck Surgery, University of Rostock Hospital, Rostock, Germany; 2 Institute of Medical Microbiology, Virology and Hygiene, University of Rostock Hospital, Rostock, Germany; 3 Department of Orthopedics, Biomechanics and Implant Technology Research Laboratory, University of Rostock Hospital, Rostock, Germany; Charité, Campus Benjamin Franklin, Germany

## Abstract

**Background:**

High hydrostatic pressure (HHP) treatment can eliminate cholesteatoma cells from explanted human ossicles prior to re-insertion. We analyzed the effects of HHP treatment on the microbial flora on ossicles and on the planktonic and biofilm states of selected isolates.

**Methodology:**

Twenty-six ossicles were explanted from cholesteatoma patients. Five ossicles were directly analyzed for microbial growth without further treatment. Fifteen ossicles were cut into two pieces. One piece was exposed to HHP of 350 MPa for 10 minutes. Both the treated and untreated (control) pieces were then assessed semi-quantitatively. Three ossicles were cut into two pieces and exposed to identical pressure conditions with or without the addition of one of two different combinations of antibiotics to the medium.

Differential effects of 10-minute in vitro exposure of planktonic and biofilm bacteria to pressures of 100 MPa, 250 MPa, 400 MPa and 540 MPa in isotonic and hypotonic media were analyzed using two patient isolates of *Staphylococcus epidermidis* and *Neisseria subflava*. Bacterial cell inactivation and biofilm destruction were assessed by colony counting and electron microscopy.

**Principal Findings:**

A variety of microorganisms were isolated from the ossicles. Irrespective of the medium, HHP treatment at 350 MPa for 10 minutes led to satisfying but incomplete inactivation especially of Gram-negative bacteria. The addition of antibiotics increased the efficacy of elimination. A comparison of HHP treatment of planktonic and biofilm cells showed that the effects of HPP were reduced by about one decadic logarithmic unit when HPP was applied to biofilms.

High hydrostatic pressure conditions that are suitable to inactivate cholesteatoma cells fail to completely sterilize ossicles even if antibiotics are added. As a result of the reduced microbial load and the viability loss of surviving bacteria, however, there is a lower risk of re-infection after re-insertion.

## Introduction

Cholesteatoma is defined as the presence of keratinizing squamous epithelium in the tympanic cavity. As a result of decreased clearance, this growth is macroscopically visible [1]–[2]. Chronic or recurrent infection of the middle ear cavity including the auditory bones (ossicles) is a common complication of cholesteatoma. A visible clinical manifestation of ear infection is otorrhea.

The initial steps in the pathogenesis of acquired middle ear cholesteatoma are still unknown and have been a matter of some controversy. Among the factors discussed are molecular dysregulation of keratinocytes and external stimulation by pro-inflammatory cytokines, growth factors and/or bacterial toxins. Inflammatory mediators such as histamine and platelet-activating factor (PAF) also appear to be involved in disease progression since they can cause eustachian tube dysfunction resulting in decreased mucociliary clearance. Another possible mechanism is that inflammatory mediators such as tumor necrosis factor alpha (TNF-α), interleukin 1 (IL-1), and PAF induce mucin secretion in the middle ear epithelium. This would lead to increased viscosity of middle ear effusions and decreased mucociliary clearance. As a result of both mechanisms, retained bacterial products such as lipopolysaccharide and cell wall fragments can cause a chronic inflammatory reaction in the middle ear cleft with continual release of cytokines and arachidonic acid metabolites resulting in further inflammation and host cell damage [3]–[4].

There is strong anatomic evidence for the presence of bacterial biofilms in experimental and human cholesteatomas. This form of organization impairs clearance since bacteria within biofilms are more resistant to host defense mechanisms and topical or systemic antibiotics [3], [5].

For this reason, chronically inflamed ossicles must often be removed during cholesteatoma surgery. Although modern allopastic implants show good biocompatibility and biostability, they cannot regularly achieve full restoration of hearing. The re-insertion of autologous ossicles is therefore a common procedure [6]–[8]. If, however, there is evidence of an easily removable coat of cholesteatoma cells or even infiltration of cholesteatoma matrix into bone tissue, ossicles should not be re-inserted [9]–[10]. The use of fixed heterologous ossicles from an ossicle bank is no longer recommended because of the potential risk of slow virus or prion transmission [11]. For this reason, devitalization of cholesteatoma cells and biofilm bacteria on ossicular surfaces should precede the re-insertion of ossicles.

Devitalization can possibly be achieved by high hydrostatic pressure (HHP) treatment. HHP can effectively disrupt or even completely destroy eukaryotic cell membranes, elements of the cytoskeleton, and enzyme systems [12]–[14]. It devitalizes bone tissue without adverse effects on the bone matrix [15]–[16]. Pressures of up to 600 MPa in particular do not alter the biomechanical properties of bone and tendon material [17]–[18].

In a previous study [19], our research group was able to show that a pressure of 400 MPa caused extensive membrane damage and thus led to a thorough inhibition of cholesteatoma cell growth on ossicles. Similar results were obtained with other types of bone [20]–[22]. In these studies, HHP treatment inactivated cells on bone surfaces without affecting rigid bone structures and thus destroyed harmful cell components on bone tissue.

The re-insertion of microbially contaminated ossicles, however, carries the risk of infectious complications. In this context, it is interesting to note that HHP can also inactivate microorganisms. In the food industry, for example, HHP has been used as a substitute for pasteurization. In this case, the level of pressure required for microbial inactivation depends on the target species [23]–[24].

Against this background, the primary aim of our study was to determine whether pressures that devitalize cholesteatoma cells can also inactivate microorganisms on ossicles. As a secondary aim, we analyzed differential effects of HHP on the planktonic and biofilms states of selected patient isolates.

## Materials and Methods

### Patients

Twenty-five patients with chronic atticoantral suppurative otitis media were recruited for this study. The inclusion criterion was a definitive need for the surgical removal of the ossicles from one middle ear cavity because of a highly destructive growth and impending complications such as hearing loss, facial nerve paresis, vertigo or hemorrhage.

### Preparation and transportation of ossicles

Tissue specimens were immediately transferred to 2 ml of a sterile 0.9% NaCl solution at a pH of 7.4. They were transported to a diagnostic laboratory within two hours after surgery and kept at a temperature of 8°C.

### Assessment of microbial colonization of patient material

Microorganisms that colonized the 5 untreated ossicles were directly processed on the basis of the standard operating procedures that are established at the routine diagnostic laboratory of the University of Rostock Hospital accredited according to DIN EN ISO 15183.

For the mobilization of biofilm bacteria, all specimens were subjected to ultrasound treatment (Sonorex 10P, Bandelin, Berlin, Germany) for 4 minutes at 80% of maximum energy in their transport media under sterile conditions prior to their transfer to culture media. Subsequently, 100 µL aliquots were spread on Columbia agar with 5% sheep blood, chocolate agar, Schaedler agar, Schaedler KV agar (supplemented with kanamycin and vancomycin) (BD), brain-heart infusion (BHI) broth, and thioglycolate broth. All media were obtained from BectonDickinson, Heidelberg, Germany. The media were incubated either in an atmosphere of 20% O_2_ and 5% CO_2_ or in an anaerobic atmosphere (Schaedler media, thioglycolate broth) at 36°C for 14 days. The media were inspected for microbial growth on days 1, 2, 4, 7 and 14. When visible growth was detected in the liquid media, aliquots were transferred to all types of agar plates and incubated as described above. For semi-quantification, growths on directly inoculated agar plates were assigned into 2 categories (few colonies = light growth, more than two dozen colonies = heavy growth). When these results were combined with growth results from liquid media, there were a total of 4 categories (“−” no growth in liquid media and on solid media; “+/−” growth only in liquid media, no growth on solid media; “+” growth in liquid media and light growth on solid media; “++” growth in liquid media and heavy growth on solid media).

After obligate anaerobic and facultative aerobic organisms had been identified, differentiation of all visible colonies was performed using commercial biochemical identification systems (VITEK 2 [bioMérieux, Nürtingen, Germany)] API® [bioMérieux] or RapID™ [remel, Thermo Fisher Scientific, Lenexa, KS, United States]). Results were confirmed for each isolate by 16S rDNA sequencing using the primers AGAGTTTGATCMTGGCTCAG and CCGTCAATTCMTTTRAGTTT (bases 1–917 of the *E. coli* 16S rDNA gene, NCBI accession no. NC_009085.1) or, if appropriate, by 18S rDNA sequencing using the primers ACTGCGAATGGCTCATTAAATCAG and CAAGGCCATGCGATTCG (bases 86–279 of the V1 region of the *C. albicans* 18S rDNA gene, GenBank accession no. AY251634).

Where possible, antibiotic resistance patterns of isolates were determined using a commercial system (VITEK 2, bioMérieux). For fastidious facultative aerobes, specific antibiotics were tested using E-test strips (AB Biodisk, Solna, Sweden) according to the standard operating procedures used at the accredited laboratory.

The differentiated strains were stored at −80°C using the Microbank Tube system according to the manufacturer's instructions (Pro-Lab Diagnostics, Round Rock, TX, United States).

The biofilm-forming capacity of all isolates was assessed by growing the strains in 96-well microtiter plates using BHI broth or thioglycolate broth for anaerobic isolates according to the protocol of Standar et al. [25]. After safranin staining, biofilm mass was measured on the basis of photometric extinction at 600 nm (OD600 nm). Previous measurements showed that OD600 nm values above 0.05 indicate the presence of a multi-layered biofilm [26]. Measurements were performed in triplicate (technical replicates) on three independent occasions (biological replicates). Biofilm formation was recorded as positive when at least two positive technical replicates were obtained on at least two occasions.

### High pressure treatment of patient material

For an assessment of the effects of high pressure treatment on human ossicles colonized by cholesteatoma cells, the bones were cut into two equally sized pieces under sterile conditions. One piece was immediately treated with high hydrostatic pressure (HHP) while the other piece was kept in a sterile humid chamber. HHP treatment was performed in closed 2.7 ml cryovials (Greiner, Nürtingen, Germany) that were completely filled with Dulbecco's modified Eagle medium (DMEM) with 10% fetal calf serum, 100 units/ml penicillin, 100 µg/ml streptomycin, and 25 µg/ml amphotericin (GIBCO, Invitrogen, Darmstadt, Germany). Care was taken to prevent bubble formation.

Pressure treatment was performed at 350 MPa for 10 minutes using a high pressure unit (HDR 100-20, Schurter-Retrofit GmbH, Königsee, Germany) at the Biomechanics and Implant Technology Research Laboratory (FORBIOMIT) of the Department of Orthopedics at the University of Rostock Hospital. After the completion of HHP treatment, the two pieces of each ossicle were analyzed for microbial colonization as described above.

For an assessment of combined effects of HHP and antimicrobial treatment, six pieces of ossicles were exposed to HHP (parameters see above) either with or without the addition of antibiotics. Three pieces were immersed in a solution containing cefuroxime 11.1 µg/ml, gentamicin 44.4 µg/ml and imipenem 3.7 µg/ml. The other three pieces were placed in a solution containing vancomycin 11.1 µg/ml, clindamycin 0.75 µg/ml and imipenem 3.7 µg/ml. After treatment, microbial colonization was again assessed as described above.

### *In vitro* studies of patient isolates

After all patient isolates were analyzed for in vitro biofilm formation in BHI broth after incubation for 3 days at 37°C and 30°C [25], [26], one patient strain of *Staphylococcus epidermidis* and one patient strain of *Neisseria subflava* that were strong biofilm formers in vitro were selected for differential pressure tests.

For an analysis of the planktonic state, *Staphylococcus epidermidis* was grown in tryptic soy broth (CASO, heipha, Eppelheim, Germany) for 24 hours and *Neisseria subflava* in BHI broth (BectonDickinson, Heidelberg, Germany) for 48 hours. Using the specific growth media, optical density was adjusted to give an extinction of 0.35 at 600 nm, which corresponded to a bacterial density of 1×10^8^ colony forming units (CFU) per ml. The cell suspensions were further diluted to 1×10^6^ CFU/ml. Aliquots of 1 ml were transferred into an appropriate number of cryovials and kept on ice until further processing.

The vials were subjected to pressures of 100, 250, 400 and 540 MPa on four independent occasions (biological replicates). After HHP treatment, bacterial suspensions were serially diluted and 100 µl aliquots were spread on tryptic soy agar (*S. epidermidis*) or BHI agar (*N. subflava*). The media were incubated in an atmosphere of 20% O_2_ and 5% CO_2_ for 24 hours (*S. epidermidis*) or 48 hours (*N. subflava*). Visible colonies were then counted. Incubation was continued for another 48 hours and colonies were counted again with a view to excluding delayed growth as a result of HHP treatment.

For an analysis of the biofilm state, *N. subflava* and *S. epidermidis* were grown and adjusted to a concentration of 1×10^6^ CFU/ml as described above. From these suspensions, 1 ml aliquots were transferred to 24-well plates containing polystyrene cover slips. The cover slips, which had a diameter of 15 mm, were cut to the size and shape required to fit the cryovials and were disinfected before being used in the experiments.

Incubation time was 3 days under the conditions described above. Then the cover slips were gently washed once with 0.9% NaCl solution to remove non-adherent cells. They were then transferred into cryovials (Greiner) that were filled with either H_2_O or 0.9% NaCl solution and kept on ice until further processing. The cryovials were subjected to HHP treatment by using pressures of 100, 250, 400, and 540 MPa on four independent occasions. After pressure treatment, the cover slips were transferred into glass tubes filled with 1 ml of phosphate buffered saline (PBS) and biofilm cells were mobilized by ultrasound treatment as described above. For comparison, untreated controls were processed in the same way, except for HHP treatment. Viable counts were performed as described for planktonic cells.

One Gram-positive strain (*Staphylococcus epidermidis*, ATCC 12228) and one Gram-negative strain (*Pseudomonas aeruginosa*, ATCC 27853) served as independent controls for planktonic cells and were subjected to the same experimental conditions, except for the pressure parameters (only pressures of 100 and 540 MPa were used).

### Electron microscopy

During every step of HHP treatment, 200 µl aliquots of planktonic patient isolates and untreated controls were taken and fixed for electron microscopy as described earlier [25]. The samples were subjected to critical point drying, sputter coated with gold [25] and documented with a scanning electron microscope (SEM) (Zeiss DSM 960A, Carl Zeiss, Jena, Germany) at 10 representative sites.

For transmission electron microscopy (TEM), the samples were washed with PBS (pH 7.4) for 24 hours and then fixed with 1% osmium tetroxide. After they were washed with PBS (pH 7.4) and dehydrated in increasing concentrations of acetone (30%, 50%, 75%, 90%), the samples were embedded in acetone/araldite. Ultrathin sections were cut on a Leica microtome Ultracut S (Leica, Solms, Germany), placed on copper grids and contrasted with lead citrate and uranyl acetate. Finally, each grid was documented at 10 representative sites using TEM (Libra 120, Carl Zeiss, Oberkochen, Germany).

### Ethics

The present study did not require ethical approval. All analyzed specimens were explanted human ossicles or parts thereof that had been irreparably damaged by cholesteatoma cells. This excess material would have otherwise been discarded without further routine analysis. Decisions in relation to surgical management were made independently of the study. Prior to the study, the authors had been informed by the ethics committee of the University of Rostock Hospital that the use of excess material required neither ethical approval nor informed patient consent. Prior to hospital admission, the patients had given general written consent for excess material to be used for research purposes. For this reason, no further informed consent was required or obtained.

## Results

### Patient data

The mean age of the patients included in this study was 37.6 years (range: 19 to 56 years). All patients showed clinical signs and audiometric test results typically associated with unilateral atticoantral suppurative otitis media. Likewise, all patients underwent middle ear surgery for the first time.

The patients' medical histories revealed that the time between onset of symptoms and definitive diagnosis ranged from 6 months to 7 years. No patient received antibiotic treatment prior to surgical intervention. No patient underwent surgery during an acute episode of the disease.

### Characterization of microbial colonization

Using established culture techniques, a total of 20 ossicles were analyzed either directly after their removal (Tables 1 and 2) or after pressure treatment (Table 2). The vast majority of ossicles were found to be colonized by microorganisms. More than one species were detected in 70% (14 of 20 ossicles) and at least one species was found in another 20% (4 of 20 ossicles). Forty-three isolates belonging to 31 bacterial species and one yeast species were differentiated. Nineteen percent (8 of 43 isolates) belonged to the group of aerotolerant anaerobes. Gram-positive species dominated with 62% (26 of 43 isolates versus 16 Gram-negative ones). The majority of isolates belonged to species currently regarded as part of the opportunistic human microflora as opposed to 3 *Pseudomonas aeruginosa* and 4 *Staphylococcus aureus* strains.

**Table 1:** Microbial colonization of untreated ossicles.

Samplenumber	Species
1	*Veilonella parvula*
	*Clostridium bifermentans*
2	*Neisseria sicca*
	*Streptococcus sanguinis* *
3	*Staphylococcus auricularis* *
	*Streptococcus mitis*
	*Neisseria subflava* * */* **
	*Proprionibacterium acnes*
	*Aeromonas salmonicida*
4	*Staphylococcus epidermidis* **
	*Ralstonia pickettii* **
	*Sphingomonas paucimobilis* *
5	*Staphylococcus hominis*
	*Sphingomonas paucimobilis* *
	*Brevundimonas diminutiva* *
	*Pseudomonas fluorescens* * */* **

All bacterial strains listed above were detected by conventional culture techniques.

*Strains that were able to form biofilms in vitro at 37°C.

**Strains that were able to form biofilms in vitro at 30°C.

**Table 2:** Microbial colonization of ossicle specimens with and without exposure to HHP treatment (350 MPa, 10 minutes).

Samplenumber	Species	Semi-quantification without pressure treatment	Semi-quantification after pressure treatment
1	*Staphylococcus capitis*	++	−
	*Neisseria subflava* * */* **	++	−
	*Candida albicans*	++	−
	*Haemophilus somnus* *	++	−
	*Burkholderia cenocepacia*	++	++
2	*Pseudomonas aeruginosa* * */* **	++	−
	*Bacteroides urealyticus*	++	−
3	*Corynebacterium pseudodiphtheriticum*	−	+
4	*Pseudomonas aeruginosa* * */* **	++	−
	*Propionibacterium acnes*	−	+
5	No bacterial growth	−	−
6	*Pseudomonas aeruginosa* * */* **	++	−
7	*Turicella otidis* *	+/−	++
	*Bacillus licheniformis*	++	+/−
8	*Eubacterium limosum*	+/−	−
9	*Propionibacterium granulosum* **	+/−	+/−
	*Propionibacterium acnes*	−	+
	*Staphylococcus hominis*	−	+
10	*Staphylococcus aureus* *	++	++
	*Staphylococcus aureus* * (morphologically distinct)	++	++
11	*Kocuria rosea* *	+/−	−
12	No bacterial growth	−	−
13	*Staphylococcus aureus*	++	++
	*Staphylococcus simulans* *	++	++
14	*Staphylococcus auricularis* *	++	−
	*Propionibacterium acnes*	+	−
15	*Staphylococcus caprae*	++	++
	*Staphylococcus aureus* *	+/−	−
	*Propionibacterium acnes*	++	−

All bacterial strains listed above were identified using semi-quantitative culture techniques. “−” No growth in liquid media and on solid media. “+/−” Growth only in liquid media. “+” Growth in liquid media plus light growth on solid media. “++” Growth in liquid media plus heavy growth on solid media;

*Strains that were able to form biofilms in vitro at 37°C.

**Strains that were able to form biofilms in vitro at 30°C.

Two of 6 ossicles subjected to pressure treatment with or without exposure to antibiotics were colonized with 7 isolates (6 Gram-positive and 1 Gram-negative isolates), two of which belonged to species that had not been identified in the previous experiments. These species too were regarded as part of the opportunistic flora.

When relative microbial quantities were assessed before HHP treatment (Table 2), 23 isolates were initially detected on native ossicles and 17 showed heavy growth.

Antibiotic resistance patterns of the isolates were determined using a commercial automated system or E-tests. The results are shown in Table S1. Except for one *S. epidermidis* strain, one *S. paucimobilis* strain and two *P. aeruginosa* strains, none of the isolates demonstrated resistance against more than two antibiotics. At least in the planktonic state, these isolates should thus be susceptible to conventional empirical antibiotic regimens.

Twenty-two (51%) of 43 isolates displayed multi-layered biofilm formation when tested in vitro (Tables 1 and 2). Strong biofilm formation was more prominent among Gram-negative (11 of 16 isolates) than among Gram-positive bacteria (11 of 26 isolates). Only 9 strains (8 Gram-negative and 1 Gram-positive strains) formed biofilms at 30°C. All but 3 biofilm-positive isolates produced biofilms at 37°C.

### Effects of pressure treatment

HHP treatment was reported to successfully kill eukaryotic cells covering ossicular surfaces [19]. The question addressed here is whether this also applies to bacteria colonizing ossicular surfaces and especially to bacteria within biofilms. For this reason, 15 ossicles were exposed to a pressure of 350 MPa for 10 minutes. Two ossicles were found to be initially sterile and 13 were colonized. All detected microbes were completely inactivated in 5 samples and relative quantities were reduced in 3 further samples. No obvious reduction of relative quantities, however, was observed in 5 samples (Table 2). When the effect was analyzed with regard to single isolates, HPP completely eliminated 14 isolates, reduced the relative quantity of 1 isolate and did not affect 8 isolates. Four isolates were detected only after HHP treatment. A possible explanation for this phenomenon may be the release of viable bacteria from biofilms as a result of this vigorous treatment.

Differences in inactivation at 350 MPa for 10 minutes were demonstrated at both the genus level – as shown for *Propionibacterium* spp. and *Staphylococcus* spp. – and the strain level within a defined species – as detected for *Propionibacterium acnes*, *Staphylococcus aureus*, *Staphylococcus auricularis* and *Straphylococcus hominis* (Table 3). Whereas HHP treatment successfully inactivated *Eubacterium limosum, Kocuria rosea*, and *Leuconostoc mesenteroides* ssp. *cremoris* (Gram-positive bacteria), *Bacteroides urealyticus, Haemophilus somnus, Neisseria subflava, Pseudomonas aeruginosa* (Gram-negative bacteria) and *Candida albicans* (a yeast strain), it failed to kill *Bacillus licheniformis, Corynebacterium pseudodiphtheriticum* and *Micrococcus luteus* (Gram-positive strains) and *Acinetobacter baumannii* and *B. cepacia* complex (Gram-negative strains) under experimental conditions (Table 3).

**Table 3:** Microbial viability of Gram-positive bacteria, Gram-negative bacteria and yeasts after HHP treatment (350 MPa, 10 minutes).

Number of isolates	Species	Semi-quantification after HHP treatment
Gram-positive bacteria		
1	*Bacillus licheniformis*	+/−
1	*Staphylococcus capitis*	−
1	*Corynebacterium pseudodiphtheriticum*	+
1	*Eubacterium limosum*	−
1	*Kocuria rosea*	−
1	*Leuconostoc mesenteroides* ssp. *cremoris*	−
1	*Micrococcus luteus*	+
2	*Propionibacterium acnes*	−
2	*Propionibacterium acnes*	+
1	*Propionibacterium granulosum*	+/−
3	*Staphylococcus aureus*	++
1	*Staphylococcus aureus*	−
1	*Staphylococcus auricularis*	−
1	*Staphylococcus auricularis*	+/−
1	*Staphylococcus capitis*	+/−
1	*Staphylococcus epidermidis*	++
1	*Staphylococcus hominis*	++
1	*Staphylococcus hominis*	+
1	*Staphylococcus simulans*	++
Gram-negative bacteria		
1	*Acinetobacter baumannii*	++
1	*Burkholderia cenocepacia*	++
1	*Bacteroides urealyticus*	−
1	*Haemophilus somnus*	−
1	*Neisseria subflava*	−
3	*Pseudomonas aeruginosa*	−
Yeasts		
1	*Candida albicans*	−

See Table 2 for an explanation of the symbols used.

An analysis of in vitro biofilm formation showed that HHP treatment successfully inactivated 9 of 15 strains that were strong biofilm formers and only 5 of 13 strains that formed no biofilm or a single biofilm layer in vitro.

### Effects of a combination of HPP and antibiotic treatment

Since HHP treatment was not sufficiently effective in one third of the treated ossicles, we combined HPP treatment with antibiotic therapy in order to investigate whether this combination treatment can enhance the disinfecting effect. For this reason, 6 ossicles were specifically exposed to HHP and a combination of 3 antibiotics. Four ossicles were found to be initially sterile. The other 2 ossicles were colonized by a total of 7 isolates, 6 of which were not affected by pressure treatment. After the addition of antibiotics, however, none of these isolates were detectable by culture techniques. Only a *Leuconostoc mesenteroides* ssp. *cremoris* strain that was not detected after HHP treatment without antibiotics grew in liquid culture after exposure to antibiotics during HHP treatment (Table 4). This particular strain was resistant to vancomycin and clindamycin. Vancomycin resistance was reported to be a general characteristic of the *Leuconostoc* genus [27].

**Table 4:** Microbial colonization of ossicles after HHP treatment (350 MPa, 10 minutes) with and without the addition of antibiotics to the media.

Samplenumber	Species	Semi-quantification after pressure treatment in the absence of antibiotics	Semi-quantification after pressure treatment in the presence of antibiotics
1	No bacterial growth	−	−
2	No bacterial growth	−	−
3	*Acinetobacter baumannii*	++	−
	*Leuconostoc mesenteroides* ssp. *cremoris*	−	+/−
4	*Staphylococcus hominis*	++	−
	*Micrococcus luteus*	+/−	−
	*Staphylococcus auricularis*	+/−	−
	*Staphylococcus capitis*	+/−	−
	*Staphylococcus epidermidis*	++	−
5	No bacterial growth	−	−
6	No bacterial growth	−	−

All bacterial strains listed above were detected by conventional culture techniques. A combination of vancomycin (11.1 µg/ml), clindamycin (0.75 µg/ml) and imipenem (3.7 µg/ml) was added to samples 1 to 3. A combination of cefuroxime (11.1 µg/ml), gentamicin (44.4 µg/ml) and imipenem (3.7 µg/ml) was added to samples 4 to 6. See Table 2 for an explanation of the symbols used.

### In vitro analysis of differential pressure effects on selected patient isolates

**Figure 1:**
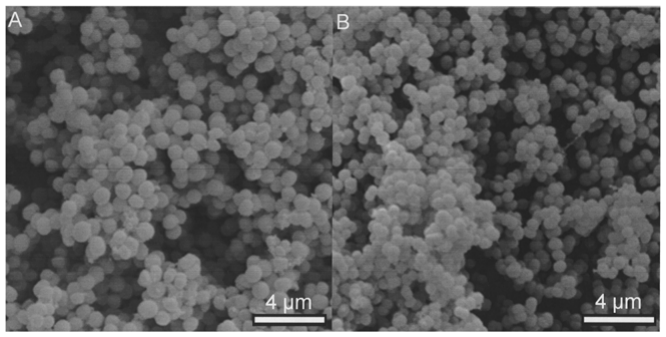
In vitro biofilms of clinical isolates before HHP treatment. Biofilms of *S. epidermidis* (A) and *N. subflava* (B) after 4 days of in vitro growth (SEM pictures, magnification ×5000).

Since the results of the ex vivo studies demonstrated varying effects of HHP treatment on Gram-positive and Gram-negative bacteria and indicated a potential influence of biofilm structures, we conducted a series of in vitro experiments to address these issues. For this purpose, an *S. epidermidis* isolate and an *N. subflava* isolate were selected (Table 1). Both isolates belonged to the majority of opportunistic isolates, displayed a normal antibiotic resistance pattern and were strong biofilm formers (Fig. 1). Since they were taken from the first group of ossicles, their susceptibility to HHP treatment had to be established. The killing effect of HHP treatment was found to be a result of cell wall damage and the destruction or alteration of cell membrane and intracellular proteins. For this reason, HHP treatment was performed in isotonic and hypotonic fluids.

**Figure 2:**
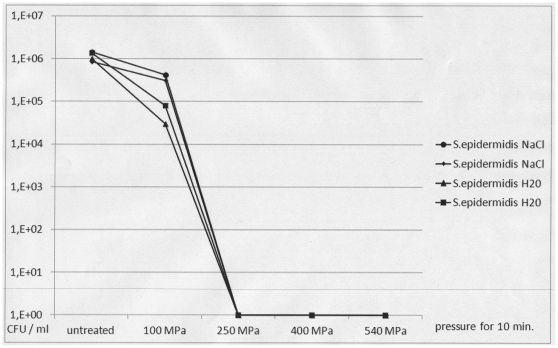
Viability of planktonic *Staphylococcus epidermidis* cells after HHP treatment using increasing levels of pressure. Pressure levels of 100 MPa, 250 MPa, 400 MPa and 540 MPa were used. For HHP treatment, planktonic *S. epidermidis* cells were suspended in 0.9% NaCl (circles and small diamonds) or H_2_O (triangles and large squares). CFU: colony forming units as determined by viability counts. The figure shows the results of two representative and independent assays.

**Figure 3:**
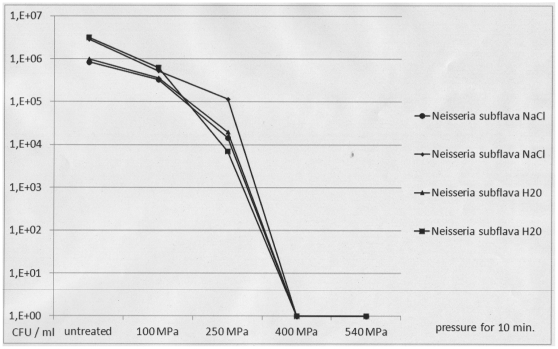
Viability of planktonic *Neisseria subflava* cells after HHP treatment using increasing levels of pressure. Pressure levels of 100 MPa, 250 MPa, 400 MPa and 540 MPa were used. For HHP treatment, planktonic *N. subflava* cells were suspended in 0.9% NaCl (circles and small diamonds) or H_2_O (triangles and large squares). CFU: colony forming units as determined by viability counts. The figure shows the results of two representative and independent assays.

**Figure 4:**
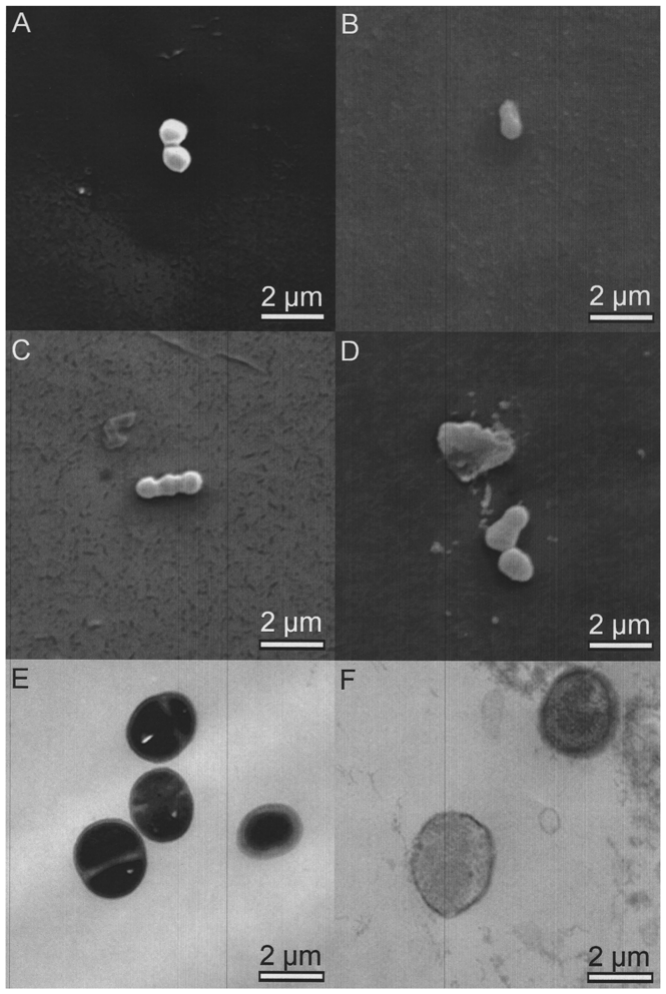
Effects of high hydrostatic pressure treatment on planktonic bacteria in isotonic medium. REM [A–D] and TEM [E, F] pictures (magnification ×10 000). *S. epidermidis* (A, B, E, F) and *N. subflava* (C, D) cells were exposed to 100 MPa for 10 minutes (A, C, E) and to 540 MPa for 10 minutes (B, D, F). Whereas cell morphology appears to be unaffected after exposure to 100 MPa, both isolates show morphological changes after exposure to 540 MPa.

When planktonic cells of both patient isolates were exposed to increasing levels of pressure, a complete decrease in viability of up to 6 decadic logarithmic units was demonstrated after 10-minute exposure to 250 MPa for *S. epidermidis* and to 400 MPa for *N. subflava* (Figs. 2 and 3). There was no difference between hydrostatic pressure treatment in hypotonic (H_2_O) and isotonic (0.9% NaCl) suspensions. Electron microscopic inspection of individual cells revealed no changes in cell shape and structure after exposure to 100 MPa for 10 minutes and substantial changes in cell shape and structure after exposure to 540 MPa for 10 minutes (Fig. 4).

Even when considerably larger amounts of planktonic bacteria (≥10^9^ CFU/ml) were exposed to the highest pressure level, survivors were observed for both the patient strains and *Staphylococcus epidermidis* ATCC 12228 and *Pseudomonas aeruginosa* ATCC 27853 controls in spite of cell damage that was visible by electron microscopy (data not shown).

**Figure 5:**
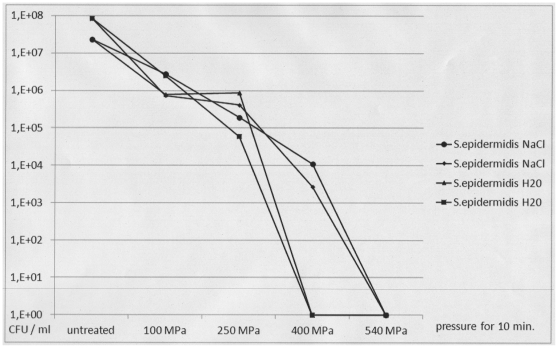
Viability of *Staphylococcus epidermidis* cells within biofilms after HHP treatment using increasing levels of pressure. Pressure levels of 100 MPa, 250 MPa, 400 MPa and 540 MPa were used. *S. epidermidis* biofilms were grown for 3 days. For HPP treatment, the cells were suspended in 0.9% NaCl (circles and small diamonds) or H_2_O (triangles and large squares). CFU: colony forming units as determined by viability counts. The figure shows the results of two representative and independent assays.

**Figure 6:**
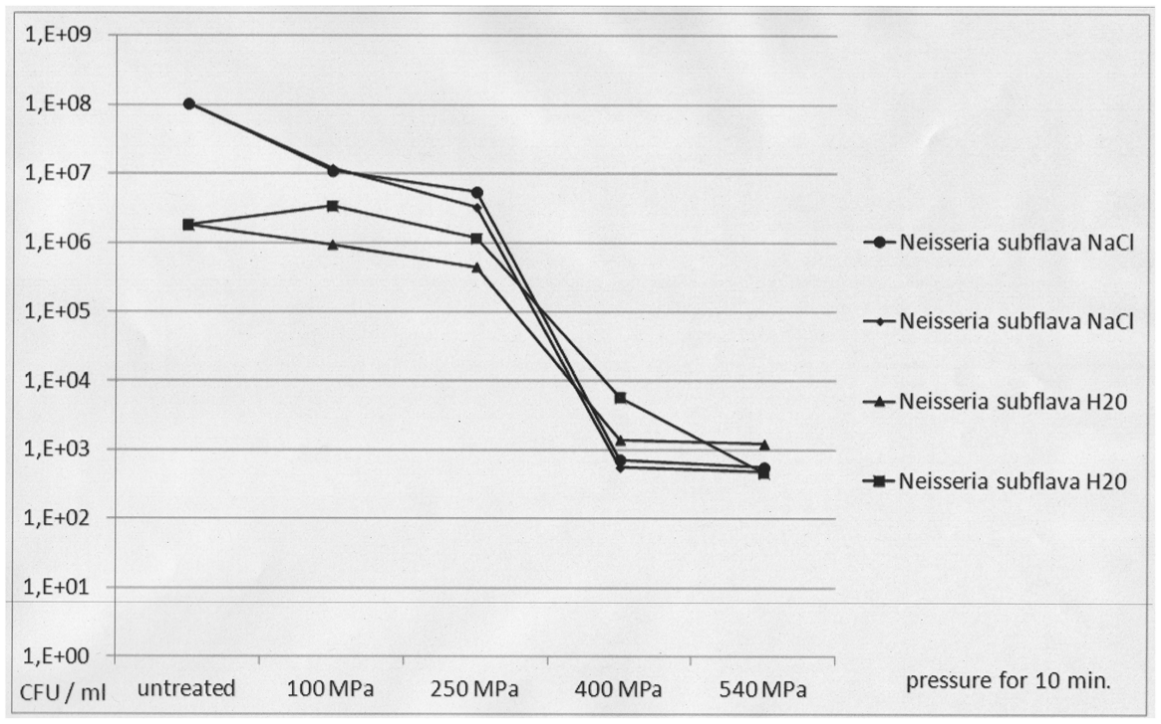
Viability of *Neisseria subflava* cells within biofilms after HHP treatment using increasing levels of pressure. Pressure levels of 100 MPa, 250 MPa, 400 MPa and 540 MPa were used. *N. subflava* biofilms were grown for 3 days. For HPP treatment, the cells were suspended in 0.9% NaCl (circles and small diamonds) or H_2_O (triangles and large squares). CFU: colony forming units as determined by viability counts. The figure shows the results of two representative and independent assays.

Under the aforementioned conditions for pressure treatment of patient strains within biofilms, inactivation of both *N. subflava* and *S. epidermidis* isolates required higher pressures. Ten minutes of exposure to at least 400 MPa were necessary to completely kill 10^7^ to 10^8^ CFU/ml of *S. epidermidis* cells in hypotonic fluid (H_2_O). Exposure to a level as high as 540 MPa for 10 minutes was necessary to inactivate a similar number of *S. epidermidis* cells in isotonic fluid (0.9% NaCl solution) (Fig. 5). *N. subflava* isolates within biofilms showed an even higher resistance to pressure treatment. Maximum pressure of 540 MPa reduced the viability count by only 3 to 5 decadic logarithmic units irrespective of biofilm immersion in hypotonic or isotonic fluid. When all parameters were taken into account, 10^3^ cells per ml survived treatment (Fig. 6).

**Figure 7:**
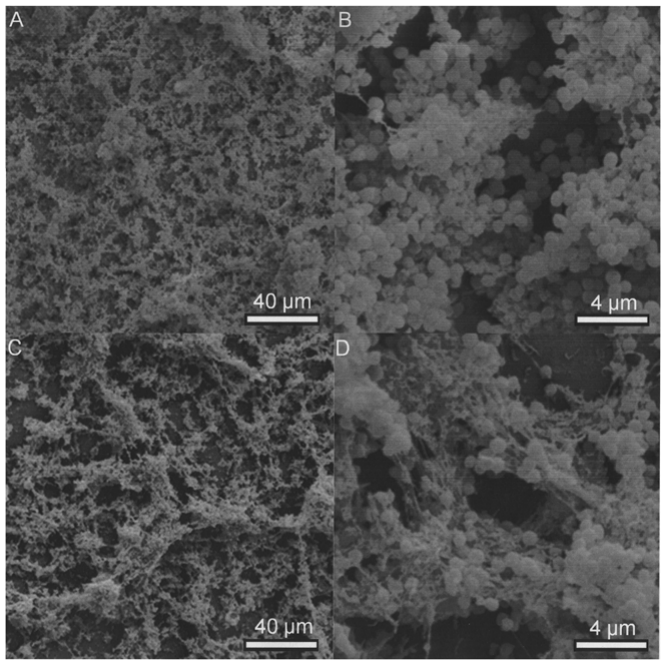
Effects of high hydrostatic pressure treatment on an in vitro *S. epidermidis* biofilm after 3 days of growth. SEM pictures. A, B: intact cell and extracellular matrix morphology before pressure treatment, magnification ×500 (A) and ×5000 (B). C, D: destruction zones with only few structurally intact cells after pressure treatment at 540 MPa for 10 minutes, magnification ×500 (C) and ×5000 (D).

**Figure 8:**
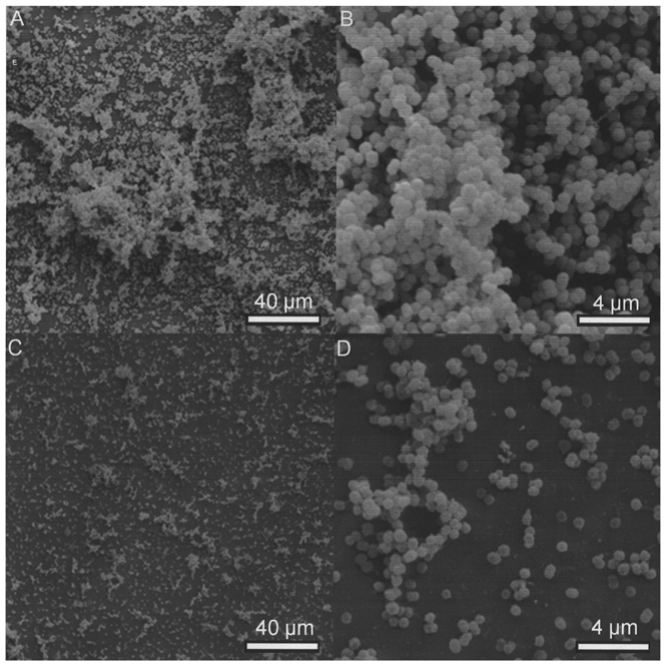
Effects of high hydrostatic pressure treatment on an in vitro *N. subflava* biofilm after 3 days of growth. SEM pictures. A, B: intact cell and extracellular matrix morphology before pressure treatment, magnification ×1000 (A) and ×5000 (B). C, D: destruction zones with only few structurally intact cells after pressure treatment at 540 MPa for 10 minutes, magnification ×1000 (C) and ×5000 (D).

Electron microscopic inspection of pressure-treated biofilms demonstrated severe damage or alteration of cells and intercellular matrix. Even at the highest pressure settings at which no viable *S. epidermidis* cells were detectable, however, small islands of normally shaped cells of both bacterial species were seen and were surrounded by severely damaged neighboring cells (Figs. 7 and 8).

## Discussion

High hydrostatic pressure (HHP) treatment has been thoroughly investigated for its ability to inactivate bacteria [23]–[24], [28] and viruses [29] in food samples. There is, however, a paucity of research addressing potential benefits of this technology in medicine. To our knowledge, the present study is the first to assess the effects of HHP treatment on microorganisms colonizing human ossicles that were obtained from cholesteatoma patients.

The study had several objectives: i) to characterize typical microbes colonizing the ossicles of cholesteatoma patients; ii) to assess whether HHP treatment can effectively remove colonizing microbes at settings previously shown to eradicate cholesteatoma cell growth on human ossicles without harming the ossicle itself [19]; iii) to investigate whether a combination of HHP and antibiotics increases the effects of treatment on colonizing microbes, and iv) to determine the potential influence of biofilm organization on microbial survival during exposure to HHP treatment.

An analysis of the microbial flora colonizing the ossicles of cholesteatoma patients revealed the presence of a broad spectrum of predominantly opportunistic Gram-positive and Gram-negative bacteria and occasional yeasts growing under aerobic and anaerobic conditions on the vast majority of the ossicles. This finding is different from a previous study, according to which the pathogen most frequently associated with cholesteatoma was *Pseudomonas aeroginosa* followed by *Staphylococcus aureus* and *Proteus mirabilis* [30]. Neither the type of patients included in the two studies nor the culture techniques can explain this difference.

When 15 ossicles from cholesteatoma patients were treated under HHP conditions that effectively inactivated cholesteatoma cells, the procedure alone disinfected less than half of the clinical samples. This result is consistent with previous studies addressing the heterogeneous susceptibility of bacteria to high hydrostatic pressure [24], [31]. It is interesting to note that we observed different inactivating effects on diverse strains of a species, for example *Propionibacterium acnes*, *Staphylococcus aureus*, *Staphylococcus auricularis* and *Staphylococcus hominis*. A possible explanation is that the pressure level was close to the threshold for inactivation of these species so that different bacterial cell numbers on the ossicles might be the reason for the different inactivation results. Another possibility is that individual strains of a species can in fact display marked differences in susceptibility during HHP exposure [32]–[33]. It is also possible that different numbers of resistant subpopulations exist within a strain [34]. Compared with Gram-negative strains, Gram-positive strains have thicker cell walls that may provide more effective protection and may explain their lower susceptibility to HHP treatment. As reported in previous studies on bone samples, *P. aeruginosa* was effectively inactivated [35] whereas the *Acinetobacter* strain with its intermediate layer of the cell wall [36] showed similar survival rates as staphylococci.

The precise mechanisms leading to HHP resistance are obviously complex and appear to vary between individual species. HHP susceptibility was found to be associated with genetic variability [37], the activation of several stress response pathways [38], the expression or hydration of macromolecules [34], [39], and the production of dysfunctional proteins [40]. In addition, assessments of the efficacy of HHP treatment can be affected by technical factors associated with microorganisms such as initial cell numbers and microbial growth phase, sublethal stress conditions prior to exposure, medium composition and culture conditions during recovery or by factors associated with the HHP protocol such as the number of compression cycles [28], [32], [41]–[48]. Our study found that osmotic pressure during HHP treatment had little or no influence (Fig. 5). It, however, cannot identify the conditions that were responsible for the variability of HHP susceptibility. This is especially due to the fact that explanted ossicles from cholesteatoma patients are rare materials so that it is impossible for us to conduct larger and more standardized experimental series.

In the few available studies on the use of HHP in medicine, this treatment was found to be similarly effective or less effective in disinfecting exposed material. When, for example, bone samples from patients with chronic osteomyelitis were subjected to HHP treatment at 600 MPa, complete disinfection was achieved in no more than 2 of 37 cases [35]. In another study investigating HPP treatment of bone samples, 71% of pressure-treated samples and 38% of untreated controls were culture-negative [49]. Complete disinfection of bone samples contaminated in vitro was achieved in about two thirds of the samples infected with *S. aureus* or *P. aeruginosa* and in 0% of the samples infected with *Enterococcus faecalis* [35].

Our findings suggest that the addition of antibiotics to the medium that is used for HHP treatment can improve the disinfection efficacy of HHP treatment. Since only three samples were used for each of the two combinations of antibiotics, however, conclusions regarding the superiority of one combination over the other cannot be drawn from this study.

One factor likely influencing the efficacy of HHP treatment is the ability of bacteria to form biofilms [37]. In our study, 51% of the strains isolated from the ossicles of cholesteatoma patients formed biofilms in vitro. This percentage indicates the potential relevance of this factor in the present study. This finding confirms the results of another study in which biofilms formed by bacteria that are occasionally present in the middle ear cavity were detected in situ or ex vivo on the ossicles of chronically infected patients [5], [50], [51] as well as on ossicular prostheses [52]–[53].

Two patient isolates identified as strong biofilm formers in vitro were exposed to HHP treatment in both their planktonic and biofilm states. A comparison of the two forms showed that the killing of biofilm cells required at least twice as much pressure as the inactivation of a similar quantity of planktonic cells. This finding applied to both Gram-positive and Gram-negative isolates. It is interesting to note that the Gram-negative bacterial species was more resistant to HHP treatment than the Gram-positive one. This suggests that cell wall thickness may play a minor role in biofilms. The complete eradication of *N. subflava* on HHP-treated ossicles (Table 2) at a much lower pressure level (350 MPa) than that required in vitro may indicate, however, that it is possible that this species does not form biofilms in vivo.

In conclusion, HHP treatment alone does not appear to be a method that can reliably and completely disinfect ossicles during middle ear surgery. It can, however, reduce the microbial load on ossicles in the majority of cases. Additional procedures such as the addition of antibiotics to the medium used during HHP treatment have the potential to improve disinfection. Although even a combination of different methods is perhaps not able to remove the entire microbial load in all cases, it may be able to reduce the bacterial load in such a way that HHP-treated ossicles can be re-inserted successfully. Compared with the hygienic requirements of bone and joint surgery [35], less demanding requirements apply to the insertion of material into non-sterile body sites such as the middle ear cavity. Unlike the hygienic requirements of bone and joint surgery [35], the physiologically non-sterile environment of the middle ear implies different minimum hygienic requirements for implant material. Further studies should be conducted to assess which combination of antibiotics is best suited to enhance the effects of HHP treatment.

## Supporting Information

Table S1 **Antibiotic resistance traits of bacterial strains isolated from ossicles of cholesteatoma patients.**
(DOC)
