# Supplementary material for: Effects of High Hydrostatic Pressure on Bacterial Growth on Human Ossicles Explanted from Cholesteatoma Patients
Source: PLoS One. 2012 Jan 23;7(1):e30150. doi: 10.1371/journal.pone.0030150 (PMC3264599; doi:10.1371/journal.pone.0030150)
Supplement: Table S1 — Antibiotic resistance traits of bacterial strains isolated from ossicles of cholesteatoma patients. (DOC) [file pone.0030150.s001.doc]

Supplemental Table S1. Antibiotic resistance traits of bacterial strains isolated from ossicles of cholesteatoma patients.

A) Test panel of antibiotics contained in VITEK2 cards.

Gram-positive bacteria Penicillin, Oxacillin, Ampicillin + Sulbactam, Cefuroxime, Imipenem; Fosfomycin; Levofloxacin, Moxifloxacin; Gentamicin, Tobramycin; Erythromycin; Tetracyclin; Sulfamethoxazole / Trimethoprim; Clindamycin; Rifampicin; Linezolid; Vancomycin, Teicoplanin; Fusidinic acid; Nitrofurantoin; Tigecycline; Mupirocin.

Gram-negative bacteria Ampicillin, Ampicillin + Sulbactam, Piperacillin, Piperacillin + Tazobactam, Cefuroxime, Cefpodoxime, Cefotaxime, Ceftazidime, Imipenem, Meropenem; Ciprofloxacin, Levofloxacin; Gentamicin, Tobramycin; Tetracyclin; Sulfamethoxazole + Trimethoprim; Nitrofurantoin.

B) Test panel of antibiotics used for E-tests.

Gram-positive bacteria Penicillin; Fosfomycin; Levofloxacin, Moxifloxacin; Gentamicin, Amikamycin; Erythromycin; Tetracyclin; Sulfamethoxazole; Clindamycin; Rifampicin; Linezolid; Vancomycin, Teicoplanin.

Gram-negative bacteria Piperacillin, Piperacillin + Tazobactam, Ceftazidime, Cefepime, Imipenem, Meropenem; Ciprofloxycin, Levofloxacin; Gentamicin, Tobramycin, Amikacin; Tetracyclin; Sulfamethoxazole; Colistin.

Yeasts Amphotericin; Flucytosin; Fluconazol, Voriconazol, Posaconazol; Caspofungin, Anidulafungin.

C) List of microbial strains resistant to ≥ 3 antibiotics from the test panels of A) and B).

*Pseudomonas aeruginosa* strain 1: Piperacillin, Piperacillin + Tazobactam, Cefepime, Ceftazidime, Imipenem, Meropenem; Gentamicin, Amikacin; Tetracyclin.

*Pseudomonas aeruginosa* strain 2: Ampicillin, Ampicillin + Sulbactam, Piperacillin, Piperacillin + Tazobactam, Cefuroxime, Cefpodoxime, Cefotaxime; Gentamicin, Tobramycin; Tetracyclin; Sulfamethoxazole + Trimethoprim; Nitrofurantoin.

*Sphingomonas paucimobilis*: Gentamicin, Tobramycin, Amikacin; Colistin.

*Staphylococcus epidermidis*: Penicillin, Oxacillin, Ampicillin + Sulbactam, Cefuroxime, Imipenem; Fosfomycin; Levofloxacin, Moxifloxacin; Erythromycin; Tetracyclin; Clindamycin.
